# Temporal variation of allergenic potential in urban parks during the vegetation period: a case study from Bratislava, Slovakia

**DOI:** 10.1007/s11356-023-31137-9

**Published:** 2023-12-05

**Authors:** Eva Zahradníková, Alena Rendeková, Jana Ščevková

**Affiliations:** Department of Botany, Faculty of Natural Sciences, Comenius University, Révová 39, 811 02 Bratislava, Slovakia

**Keywords:** Polinosis, Allergenic flora, Allergenicity index, Public greenery, Pollination period, Air pollutants, Urban environment

## Abstract

Park greenery represents an oasis for urban residents; however, during the flowering period of trees that produce allergenic pollen grains, these areas threaten individuals suffering from seasonal allergic respiratory diseases. In this study, we evaluated the temporal distribution of the allergenic potential of three most important urban parks in Bratislava over the vegetation period, using a modification of the Urban Green Zone Allergenicity Index (*I*_UGZA_) and Individual-Specific Allergenic Potential Index (*I*_ISA_) designed as a running index — *rI*_UGZA_ and *rI*_ISA_. We found that *rI*_UGZA_ gives better information for park management and revitalization, since it considers the potential size of woody plants, while *rI*_ISA_, considering the actual size of the vegetation, provides more relevant information for pollen-allergy sufferers. Based on *rI*_ISA_, the allergenic potential was highest in May for the Grassalkovich Garden (formal baroque garden) and Janko Kráľ Park (English landscape park) and in April for the Medic Garden (repurposed baroque garden). We also found differences in the duration of the period of increased allergenic potential in these parks, ranging from 1 to 3 months. Based on the total annual sums of *rI*_ISA_, we found the highest allergenic potential in the Medic Garden and lowest in the Janko Kráľ Park. This variance is caused mainly by the different density of trees and percentage of allergenic species. The biggest contributors to the allergenic potential were *Platanus*, *Acer* and *Tilia*. Based on the information on temporal variation of the allergenic potential during the vegetation period provided by the running indices, it is possible to improve the planning of park revitalization based on the flowering period of allergenic species and provide better information to the pollen-allergy sufferers for minimizing the allergenic effect of urban green areas on their health during a particular month.

## Introduction

Green spaces are a desirable component of the urban environment (Kothencz et al. 2017), but can also harm human health as green spaces are sources of allergenic pollen grains (Cariñanos et al. 2019). Allergic diseases are rising globally, with a vast clinical and economic impact. The World Health Organization (WHO) estimates the number of cases of allergic asthma to be 300 million and allergic rhinitis to be 400 million (Bousquet and Kaltaev 2007). The total cost of treatment of allergic diseases in the European Union stands between 55 and 151 billion EUR annually (Zuberbier et al. 2014).

Allergenic airborne bioparticles, often embodied in long-range atmospheric transmission, are in urban environments subject to long-term monitoring by a network of aerobiological stations throughout Europe (Buters et al. 2018). However, a specific feature of larger green areas with a significant tree cover, such as urban parks, is the trapping of different air pollutants including pollen, so that the concentration of these particles in such an area may differ profoundly from the outdoor situation (Escobedo et al. 2011). Most of the allergenic pollen grains in this area originate from local vegetation, primarily trees and grasses. Therefore, pollen concentrations provided by a network of aerobiological stations cannot be considered an appropriate proxy for the allergenic potential of urban parks. Depending on the phenophase, the allergenic potential of urban parks may be lower or higher than their surroundings.

The allergenic potential of different green areas in the urban environment can be quantified using the Urban Green Zone Allergenicity Index (*I*_UGZA_) (Cariñanos et al. 2014), firstly used in Spain to assess allergenic potential of different urban parks. This method has so far been used to calculate the allergenic potential of urban greenery principally in areas with Mediterranean climate in Spain (Cariñanos et al. 2016, 2017, Cariñanos and Marinangeli 2021; Velasco-Jiménez et al. 2020; Sabariego et al. 2021), Italy and Portugal (Cariñanos et al. 2019; Suanno et al. 2021) and Turkey (Kara and Aşık 2022). In temperate regions, it has only been applied less often, e.g. in Poland (Kasprzyk et al. 2019) and Germany (Jochner-Oette et al. 2018). Jochner-Oette et al. (2018) also used a method taking into account the actual measurements of the trees instead of their potential size, creating the Individual-Specific Allergenic Potential Index (*I*_ISA_).

The problem with insufficient green areas in the city centres is common for many cities, including Bratislava (Belčáková et al. 2022). The oldest parts of these cities follow the original building plan from centuries ago, leaving no space for new green areas, while the plots in the newer parts of the city centres are highly sought after by investors and building parks here is economically less profitable (Winkler et al. 2023). Often the only type of greenery in these areas are historical parks and gardens, from which some could be kept in their original state while others were modernised. With the increase of sedentary lifestyle, the function of urban parks as areas of active recreation is getting more important (Zhang and Zhou 2018). Paths are used by runners and bigger grassy areas can serve for sports like yoga, martial arts or badminton. Even children need more physical activity in their schedule, and playgrounds in parks can fulfil this need (Floyd et al. 2011). Connected to this is the need of expressing the changes in the allergenic potential of these spaces continuously throughout the vegetation season so that the health benefits of outdoor activity are not negated by the allergenicity of the area. Individuals sensitive to respiratory allergic diseases need this information for making a decision about the most suitable place for their recreational activity, and the park management can use it to issue warnings and recommendations as well as planning to avoid peak in allergenicity that may be undetectable by *I*_UGZA_ or *I*_ISA_. For example, a park with a low number of allergenic species can have a low value of *I*_UGZA_/*I*_ISA_ and still be highly allergenic at a particular time of the year due to the simultaneous flowering of some of these species. For this reason, we adjusted both of these indices to create a running index offering the information about the changes of the allergenic potential of these parks through the whole vegetation season.

The aim of this study was to determine the temporal variation of allergenic potential in the three most important urban parks in the city centre of Bratislava during the vegetation season, using running indices based on *I*_UGZA_ and *I*_ISA_. Additionally, we identified the woody plant species that contribute the most to the allergenic potential of these areas and proposed measures to mitigate the negative impact of these spaces on individuals with pollen allergies.

## Materials and methods

### Study area

**Fig. 1:**
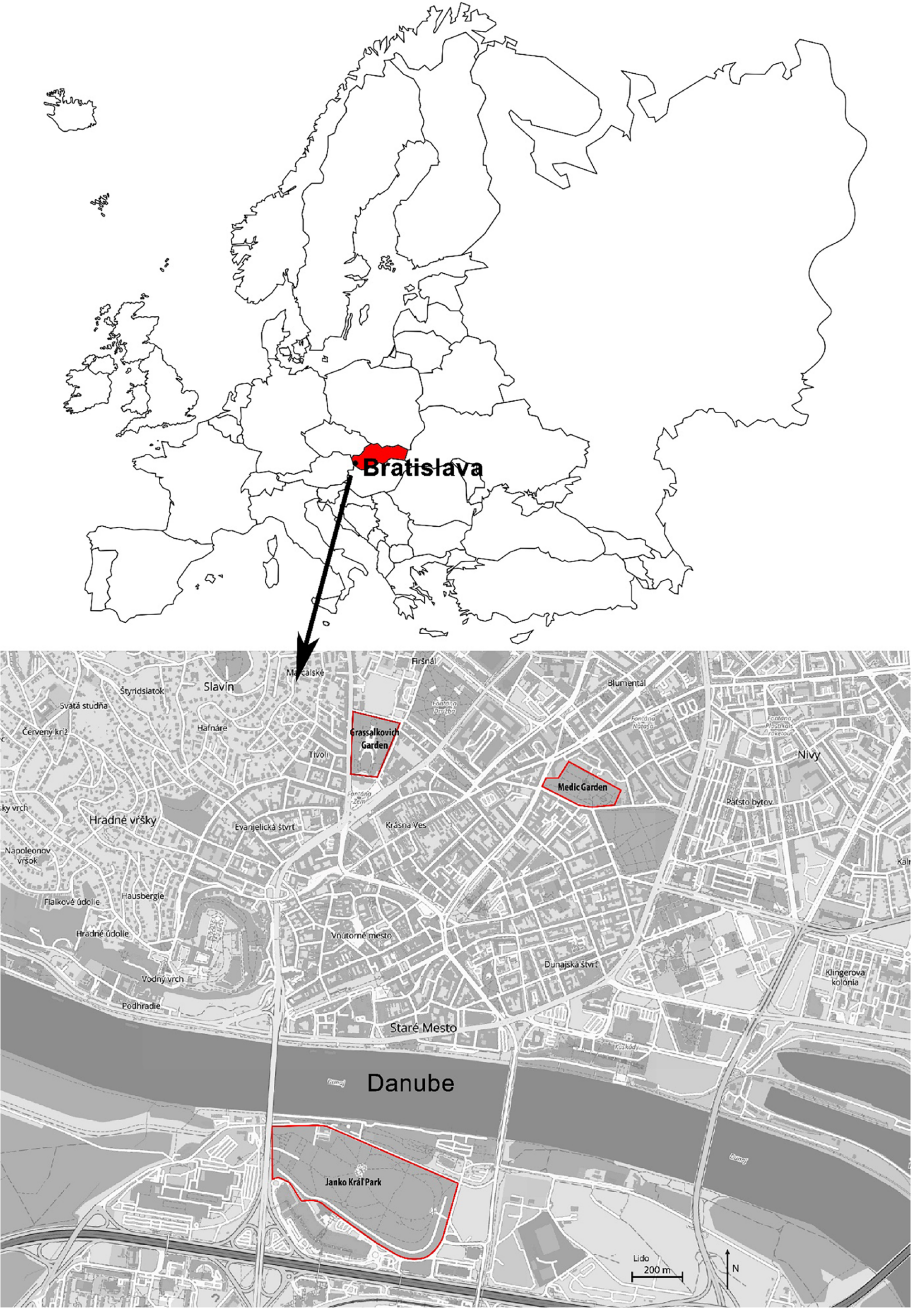
The position of the studied public parks in the city centre of Bratislava, Slovakia (source: https://mu-basm.gisplan.sk/mapa/historia)

The three following public parks in the city centre of Bratislava (Fig. 1) were picked as the study locations.

Medická záhrada (MZ, Medic Garden) was founded in 1770 as a baroque garden, but nowadays almost nothing is left from the original historical disposition after a last major reconstruction in 1985 (Steinhübel 1990). It is surrounded by the highly urbanised area of the city centre, and as such has a high number of visitors seeking its recreational function, which it was unable to satisfy in its historical shape. This led to a radical reconstruction, although some trees were left from the original disposition. It contains a more formal part with aesthetic purpose centred around a fountain, shaded lanes with trees, free grassy areas and a playground for children. In our study, it represents historical greenery repurposed into a more modern form in late twentieth century. It has an area of 3.14 ha, and the GPS coordinates are 48° 08′ 59.4″ N 17° 07′ 10.4″ E.

Sad Janka Kráľa (SJK, Janko Kráľ Park, literally Orchard) is the oldest public park in Central Europe, founded in the 1770s. It is situated on the right shore of the Danube River in the Petržalka City part. Paths were cut in the original floodplain forest and planted by alleys in the shape of an eight-rayed star. Its current disposition dates back to a reconstruction starting in 1832 in the style of an English landscape park, showcasing several newly introduced species of trees and shrubs (Steinhübel 1990). Until present, it remains one of the most important recreational green areas in Bratislava. Its area is 23 ha, with GPS coordinates 48° 08′ 05.4″ N 17° 06′ 34.6″ E.

Grasalkovičova záhrada (GZ, Grassalkovich Garden) is a baroque garden belonging to a palace that is now the seat of the president of Slovak republic, known as the Presidential Palace. The palace was built in 1760 by a Hungarian aristocrat Anton Grassalkovich (Tomaško 2004). The current disposition of the garden mostly corresponds with the historical one, with a regular parterre typical for French gardens. It is located in the urbanised area of the city centre, but the representative function it still has prevented any major changes in its disposition towards a less formal one. It is open for public but surrounded by a high solid wall that can present a psychological barrier from entering it for casual recreation. The only exception from the historical shape are wooden playground elements placed in the garden. The last reconstruction of the park was in the 1990s. The area of the garden is 3.68 ha, and the GPS coordinates are 48° 09′ 01.3″ N, 17° 06′ 28.6″ E.

### Dendrological inventory

Trees and shrubs in all parks were determined at the level of genus or species, and their phenophases were observed in the vegetation period of 2021 and 2022. In dioecious species, only male individuals were counted. Measurements were taken at the same time: crown diameter and height (m) for trees and height (m) and ground coverage (m^2^) for shrubs. Using these data, we calculated the crown volume of each specimen using the formula for an ellipsoid (broad-leaved species) or a cone (conifers). We did not consider the volume of the grassy areas since the mowing regime is the same in all studied localities and aims to prevent their flowering and emission of pollen as much as possible. The nomenclature of taxa and syntaxa are based on WFO (2023).

### Calculating allergenic potential

In *I*_UGZA_ (Cariñanos et al. 2014), the allergenic potential is expressed on a scale from 0 to 1, where 1 represents the highest possible potential allergenic value (*PAV*_max_) of a given area. The value of this index is a sum of the contributions of all species in the studied area and depends from their allergenic potential (*ap*), type of pollination (*tp*), the length of the pollen production period (*ppp*) as per Table 1 and the potential volume of an individual of each species (*V*), determined by its height and crown diameter at maturity multiplied by the number of individuals of the species in the studied area (*n*). It is compared to a potential area with maximal allergenic value and maximal height of vegetation cover (*H*_max_) in the whole area (*S*_*T*_) of the studied locality. The resulting formula is:

**Table 1:** The parameters of* I*_UGZA_ according to Cariñanos et al. (2014)

Parameter	Value	Description
Type of pollination (*tp*)	0	No pollen emission (sterile, cleistogamous, female)
	1	Entomophilous
	2	Amphiphilous
	3	Anemophilous
Principal pollination period (*ppp*)	1	1–3 weeks
	2	4–6 weeks
	3	6+ weeks
Allergenic potential (*ap*)	0	Non-allergenic or not reported as allergenic
	1	Low
	2	Moderate
	3	High
	4	Main local allergens

$$\mathrm{\textit{PAV}}=tp\times ppp\times ap$$

$${I}_{\mathrm{UGZA}}=\frac{1}{{\mathrm{\textit{PAV}}}_{\mathrm{max}}\times {H}_{\mathrm{max}}\times {S}_{T}}\times \sum_{i=1}^{k}{n}_{i}\times {\mathrm{\textit{PAV}}}_{i}\times {V}_{i}$$

*I*_ISA_ uses the same method of calculation, but instead of the potential volume of an individual of each species uses actual measurements of the dendroflora in the studied area and determines its real volume.

Both indices depend on the maximum height of the vegetation in the study area, which might differ in various climatic conditions. We used the maximum height of 30 m, which corresponds to the height of several broad-leaved native species in ideal conditions.

The parameters for determining the *PAV* in both indices are the type of pollination, duration of the pollination period and allergenic potential. The increase in any of the parameters by just 1 can double the resulting *PAV*, so the border cases need to be considered carefully.

The allergenic potential of each species depends on the frequency and severity of pollinosis caused by pollen grains of that species and is available in databases and literature sources. However, these sources are only applicable to a particular region, e.g. Cariñanos and Marinangeli (2021) for the Mediterranean area, since the concentration and repeated exposure to different allergens can influence the severity of pollinosis. For a species to be considered allergenic, it must produce a high quantity of airborne pollen containing allergenic molecules, but also be abundant in the region (Hrubiško 1998). We used data considering pollen-related allergic aggressiveness of local woody plant species (Jurko 1990a, b, c; Hrubiško 1998) to determine the allergenic potential of the air in the studied area. Only when not available did we use data from as geographically and climatically close regions as possible (Jochner-Oette et al. 2018; Kasprzyk et al. 2019).

We consider the principal pollination period parameter the most unreliable of the ones used to calculate *PAV.* The difference of just a few days can double the *PAV* here, while most sources only give the flowering period in months, e.g. March–April. The solution of this problem could be using palynological data from local sources. However, as a provider of such data, we noticed big variation in the duration of the pollination period between individual years (Ščevková et al. 2010). For example, the pollination season for the genus *Populus* lasted 60 days in 2008, but only 17 days in 2006. The timing of pollen release in the atmosphere depends strongly on the weather, especially on the temperature and humidity (Bartková-Ščevková 2003), and can drastically differ between years. Other problems with using aeropalynological data is the absence of species with biotic pollination from them, and the inability to distinguish between individual species of a genus or even a family or group of families like Cupressaceae–Taxaceae.

With regard to these limitations of determining the principal pollination period and low information value of single-number indices for the general public (they are useful for planning of urban vegetation but not for informing people with pollinosis about the time window when it is safe or not safe for them to visit a particular public park), we propose using a Running *I*_UGZA_ (*rI*_UGZA_) and Running *I*_ISA_ (*rI*_ISA_) instead.

*rI*_UGZA_ and *rI*_ISA_ are calculated similarly to *I*_UGZA_ and *I*_ISA_, with omission of *ppp* from the formula. Instead, they are calculated separately for every month, using the value 1 instead of *ppp* if the species is flowering during that time and 0 if it is not. The overall allergenicity index for the studied location can be calculated as a sum of *rI*_UGZA_ or *rI*_ISA_ values for all months of the year. Therefore, our *PAV* value is calculated by a slightly different formula, using *cmf* (calendar months of flowering):

$$PAV^{\prime}=tp\times ap\times cmf$$

To mark the distinction, we mark it with an apostrophe. Since the usual flowering period is between 1 and 3 months, the values are comparable with the original *I*_UGZA_ and *I*_ISA_, which also uses values between 1 and 3 for the *ppp* parameter, therefore we mark them as *I*_UGZA_′ and *I*_ISA_′. They also correspond quite well to the original values since the flowering time of 4–6 weeks (*ppp* 2) means the plant flowers over the course of 2 calendar months. Unlike *ppp*, *cmf* is easier to find in guides to local flora (for native species) and ornamental plant catalogues (for introduced ornamental species) and does not have such a big variability between years as *ppp*.

The calculated values of *I*_UGZA_′ and *I*_ISA_′ were compared with various parameters of the parks (area, number of woody plants, their growth forms, density of trees and Shannon’s diversity index; Shannon and Weaver 1949). The values of *rI*_UGZA_ and *rI*_ISA_ for each month were compared with the parameters changing in time (the number of flowering woody plants, trees, shrubs, allergenic and wind pollinated woody plants in each month as well as the number of their species) by Spearman non-parametric test.

## Results

**Fig. 2:**
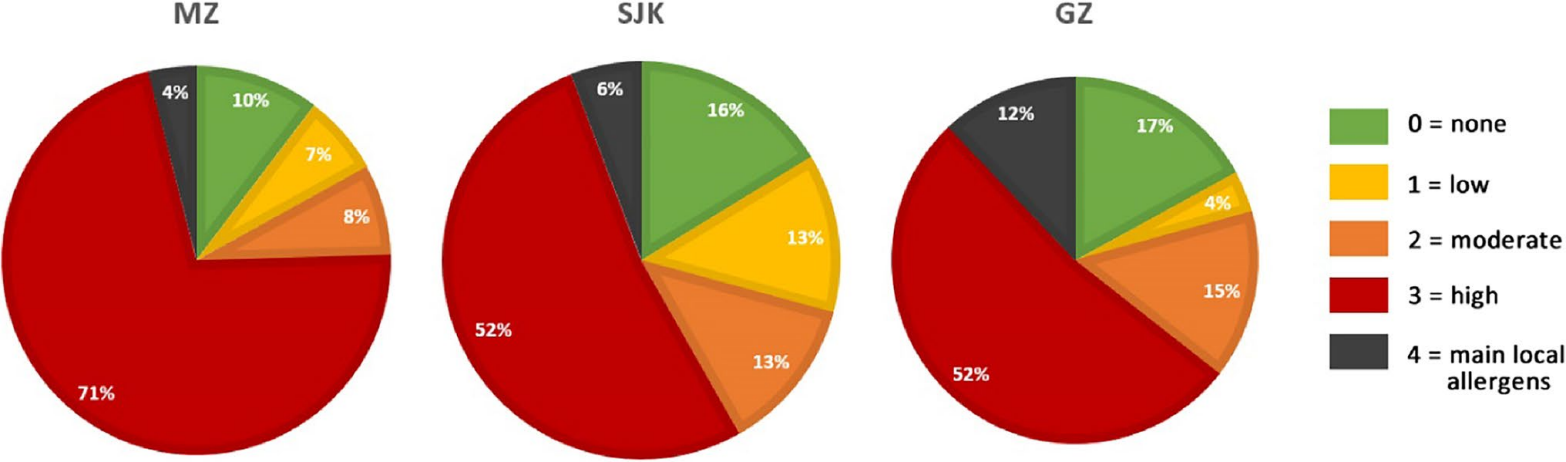
The allergenic potential of the woody plants in the studied parks: MZ — Medic Garden (327 woody plants), SJK — Janko Kráľ Park (890 woody plants) and GZ — Grassalkovich Garden (323 woody plants)

The species identified in the studied locations with their allergenic potential (*ap*), type of pollination (*tp*), flowering time and the resulting potential allergenic value (PAV′) are listed in Table 2. From the 327 woody plants found in MZ, 75% are highly allergenic (*ap* = 3–4). In SJK, it is 57% of the 890 woody plants and in GZ 64% of 323 (Fig. 2). The taxa with the highest *PAV*′ (27–36) are *Celtis occidentalis*, *Platanus* × *hispanica* and the species from the Cupressaceae family. The most abundant genera are *Tilia* (*PAV*′ = 6–12) in both MZ (36.7%) and GZ (19.57%) and *Acer* (*PAV*′ = 12) in SJK (49.85%).

**Table 2:** The species of woody plants and their numbers identified at the locations MZ — Medic Garden, SJK — Janko Kráľ Park and GZ — Grassalkovich Garden in Bratislava with their type of pollination (*tp*), allergenic potential (*ap*), flowering in calendar months and potential allergenic value (*PAV*′) valid for the area of Slovakia (Central Europe)

Species	Family	MZ	SJK	GZ	*tp*	*ap*	Flowering	*PAV'*
*Abies* sp. L.	Pinaceae		7	2	3	1	V–VI	6
*Acer saccharinum* L.	Sapindaceae	3			2	3	III–IV	12
*Acer* sp. L.	Sapindaceae	26	163	16	2	3	IV–V	12
*Aesculus hippocastanum* L.	Sapindaceae	12	59	43	2	3	V	6
*Ailanthus altissima* (Mill.) Swingle	Simaraubaceae		4	6	2	3	V	6
*Berberis julianae* C.K. Schneid.	Berberidaceae	14	5		1	1	IV–VI	3
*Berberis thunbergii* DC.	Berberidaceae		5	3	1	1	V–VI	2
*Buxus sempervirens* L.	Buxaceae	10	2	33	1	2	III–IV	4
*Carpinus betulus* L.	Betulaceae	2	13	1	3	4	IV–V	24
*Caryopteris* × *clandonensis* Simmonds	Lamiaceae			2	1	0	VII–X	0
*Castanea sativa* Mill.	Fagaceae		7		2	3	VI–VII	12
*Catalpa bignonioides* Walter	Bignoniaceae	2	7		1	0	VI–VII	0
*Celtis occidentalis* L.	Cannabaceae	15	13	2	3	3	III–V	27
*Cornus alba* L.	Cornaceae		10		1	0	V–VI	0
*Corylus avellana* L.	Betulaceae	1			1	4	II–IV	12
*Corylus colurna* L.	Betulaceae	3	9		1	4	III–IV	8
*Cotoneaster* sp. Medik.	Rosaceae		5	5	1	0	V–VI	0
*Crataegus* sp. L.	Rosaceae	2	5		1	0	V–VI	0
*Cryptomeria japonica* (Thunb. ex L.f.) D.Don	Cupressaceae		4		3	3	II–V	36
*Euonymus fortunei* (Turcz.) Hand.-Mazz.	Celastraceae			3	1	0	VI–VII	0
*Fagus sylvatica* L.	Fagaceae	6	8	2	3	4	IV–V	24
*Forsythia* × *intermedia* Zabel	Oleaceae	2	4		1	2	IV	2
*Frangula alnus* Mill.	Rhamnaceae		12		1	1	V–VI	2
*Fraxinus americana* L.	Oleaceae	1	49		3	1	IV–V	6
*Fraxinus excelsior* L.	Oleaceae	3		1	3	1	V	3
*Ginkgo biloba* L. (male)	Ginkgoaceae		1		3	0	IV–V	0
*Gleditschia triacanthos* L.	Fabaceae	1	7		1	0	V	0
*Chaenomeles speciosa* (Sweet) Nakai	Rosaceae		9		1	0	III–IV	0
*Chamaecyparis* sp*.* Spach	Cupressaceae	4	3		3	3	IV–V	18
*Ilex aquifolium* Lour.	Aquifoliaceae		3		1	0	V–VI	0
*Juglans regia* L.	Juglandaceae		11	1	3	2	IV–V	12
*Juniperus* × *media* V.D.Dmitriev	Cupressaceae		6		3	3	III–V	27
*Juniperus horizontalis* Moench	Cupressaceae	5			3	3	III–V	27
*Ligustrum vulgare* L.	Oleaceae	1	9		2	2	VI–VII	8
*Linnaea amabilis* (Graebn.) Christenh.	Caprifoliaceae	1			1	0	V–VI	0
*Liquidambar styraciflua* L.	Altingiaceae		12		3	2	III–V	18
*Liriodendron tulipifera* L.	Magnoliaceae		2		1	1	V–VII	3
*Lonicera fragrantissima* Lindl. & Paxton	Caprifoliaceae		2		1	0	I–II	0
*Lonicera nigra* L.	Caprifoliaceae	2			1	0	IV–V	0
*Lonicera pileata* Oliv.	Caprifoliaceae		2		1	0	V	0
*Lonicera tatarica* L.	Caprifoliaceae		2		1	0	V–VI	0
*Maclura pomifera* (Raf.) C.K.Schneid.	Moraceae		8		3	0	V–VI	0
*Magnolia* × *soulangeana* Soul.-Bod.	Magnoliaceae	5	2	1	1	2	III–V	6
*Magnolia tripetala* L.	Magnoliaceae			1	1	2	V–VI	4
*Mahonia aquifolium* (Pursh) Nutt.	Berberidaceae	9			1	0	IV–V	0
*Malus* sp. Mill.	Rosaceae			23	1	0	IV–V	0
*Metasequoia glyptostroboides* Hu & W.C.Cheng	Cupressaceae		8		3	3	III	9
*Morus alba* L.	Moraceae		5		3	3	V–VI	18
*Paulownia tomentosa* Steud.	Paulowniaceae		3		1	0	V	0
*Philadelphus coronarius* L.	Hydrangeaceae		2		1	3	VI–VII	6
*Physocarpus opulifolius* Raf.	Rosaceae		4		1	0	V–VI	0
*Picea pungens* Engelm.	Pinaceae	2	17	4	3	1	V–VI	6
*Pinus* sp. L.	Pinaceae		37	10	3	2	V–VI	12
*Platanus* × *hispanica* Münchh.	Platanaceae	15	65	2	3	3	IV–VI	27
*Platycladus orientalis* (L.) Franco	Cupressaceae	4	8		3	3	III–V	27
*Populus alba* L.	Salicaceae		22		3	3	III–IV	18
*Populus nigra* L.	Salicaceae	1		8	3	3	III–IV	18
*Prunus* sp. L.	Rosaceae	6	32	10	1	0	IV–V	0
*Pseudotsuga menziesii* (Mirb.) Franco	Pinaceae		3		1	1	V–VI	2
*Pyracantha coccinea* M.Roem.	Rosaceae		5	8	1	0	V–VI	0
*Quercus robur* L.	Fagaceae	1	21	36	3	4	V	12
*Rhamnus cathartica* L. (male)	Rhamnaceae		4		1	0	V–VI	0
*Ribes* sp. L.	Grossulariaceae		4		1	0	IV–V	0
*Robinia pseudoacacia* L.	Fabaceae	4	3		1	3	VI	3
*Rosa sp.* L.	Rosaceae	2	15	2	1	1	VI–VIII	3
*Salix alba* L.	Salicaceae	1	6		3	3	IV–V	18
*Sambucus nigra* L.	Adoxaceae	3	20	2	1	2	VI–VII	4
*Skimmia japonica* Thunb. (male)	Rutaceae		11		1	0	IV	0
*Spiraea douglasii* Hook.	Rosaceae		7		1	0	VI–IX	0
*Spiraea vanhouttei* (Briot) Zabel	Rosaceae	1		3	1	0	V–VII	0
*Styphnolobium japonicum* (L.) Schott	Fabaceae	2	7		2	2	VII–VIII	8
*Symphoricarpos albus* (L.) S.F.Blake	Caprifoliaceae	1			1	0	VI–VII	0
*Syringa* sp. L.	Oleaceae	2	8		1	2	V	2
*Taxus baccata* L. (male)	Taxaceae	12	9	26	3	3	III–IV	18
*Thuja occidentalis* L.	Cupressaceae	9		2	3	3	III–V	27
*Thuja plicata* Donn ex D.Don	Cupressaceae		4		3	3	III–V	27
*Tilia* × *europaea* L.	Malvaceae			64	2	3	VI–VII	12
*Tilia cordata* Mill.	Malvaceae	13	32		2	3	VII	6
*Tilia platyphyllos* Scop.	Malvaceae	107	27		2	3	VI	6
*Tilia tomentosa* Moench	Malvaceae	1			2	3	VII	6
*Ulmus glabra* Mill.	Ulmaceae		17		3	3	III–V	27
*Ulmus minor* Mill.	Ulmaceae	4			3	3	III–IV	18
*Viburnum rhytidophyllum* Hemsl.	Adoxaceae	6	14		1	0	V–VI	0
*Weigela florida* (Bunge) A.DC.	Caprifoliaceae			1	1	0	V–VI	0

**Fig. 3:**
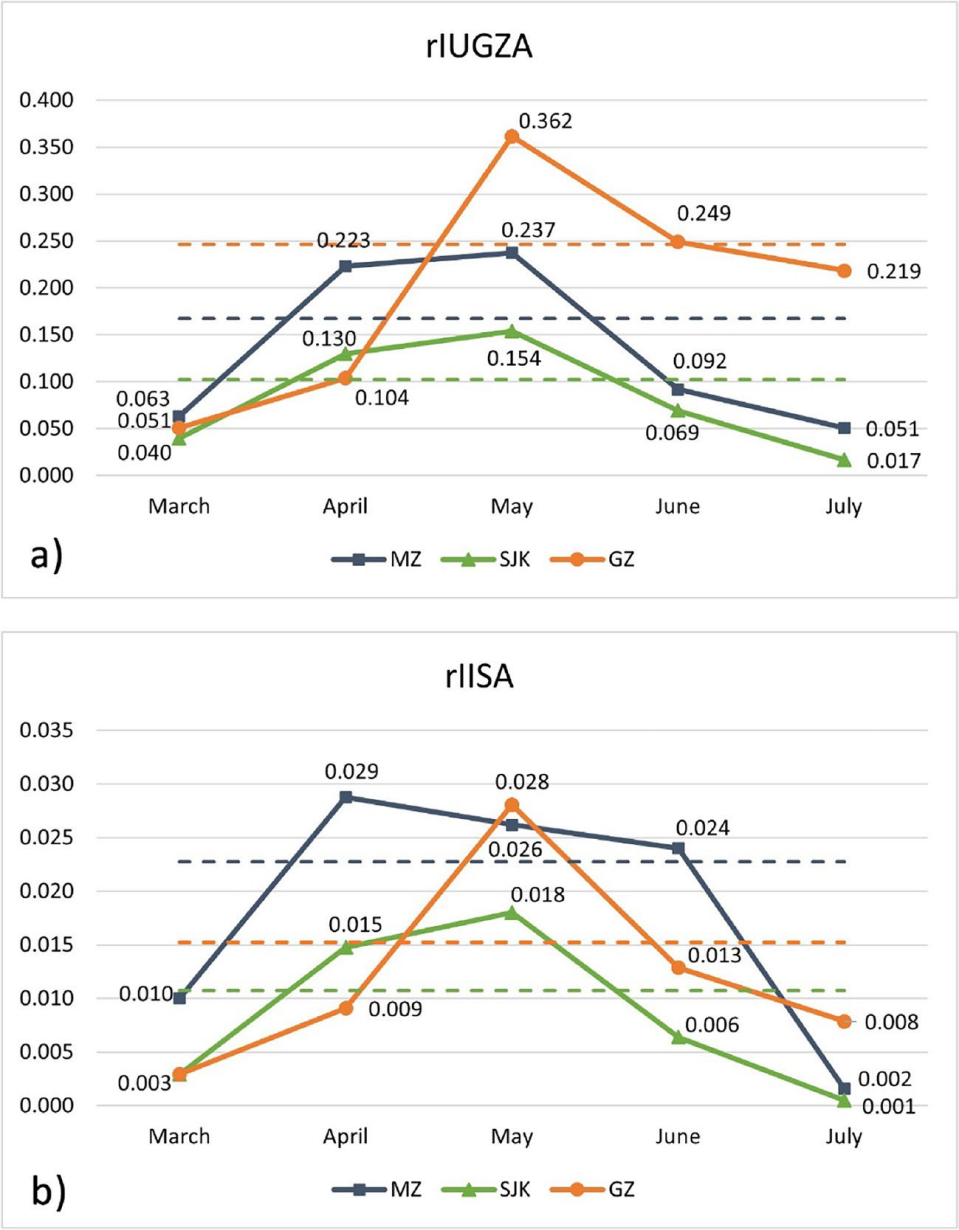
*rI*_UGZA_ (**a**) and *rI*_ISA_ (**b**) of the three studied public parks in Bratislava: MZ — Medic Garden, SJK — Janko Kráľ Park and GZ — Grassalkovich Garden, March‒July (the values are negligible for the rest of the year). The dashed lines represent 25% of the total *I*_UGZA_′ and *I*_ISA_′ value for the individual parks

Figure 3 shows the calculated values of the *rI*_UGZA_ and *rI*_ISA_ for the three studied public parks over the vegetation period, which in Bratislava lasts from February to October. However, all values were negligible before March and after July. The difference between these values is caused by the fact that *rI*_UGZA_ takes into account the potential size of the woody plants at their maturity, while *rI*_ISA_ works with their actual size at present. Therefore, *rI*_UGZA_ is better suited for long-term planning of park management and revitalization, while *rI*_ISA_ provides more relevant information about the actual situation during the vegetation season for pollen-allergy sufferers. The data show the main allergenic season in these parks lasting from March to July. For the rest of the year, the values are negligible for both *rI*_UGZA_ and *rI*_ISA_. The highest value of *rI*_UGZA_ (0.362) was noted in May in GZ and the highest value of *rI*_ISA_ (0.029) in April in MZ. The sum of the monthly indices gives the value of *I*_UGZA_′ and *I*_ISA_′ for these locations. For MZ, *I*_UGZA_′ is 0.67 and *I*_ISA_′ 0.091. SJK has *I*_UGZA_′ 0.41 and *I*_ISA_′ 0.043 and GZ has *I*_UGZA_′ 0.985 and *I*_ISA_′ 0.061. The graph also gives information about the duration of the period of increased allergenic potential in the individual parks, defined as the period when the value of *rI*_UGZA_/*rI*_ISA_ reaches over 25% of the total *I*_UGZA_′/*I*_ISA_′ over the whole vegetation period. Taking into account the value of *rI*_UGZA_, this period is April–May in both MZ and SJK and May–June in GZ. For *rI*_ISA_, it lasts from April to June in MZ, April–May in SJK and May in GZ.

**Fig. 4:**
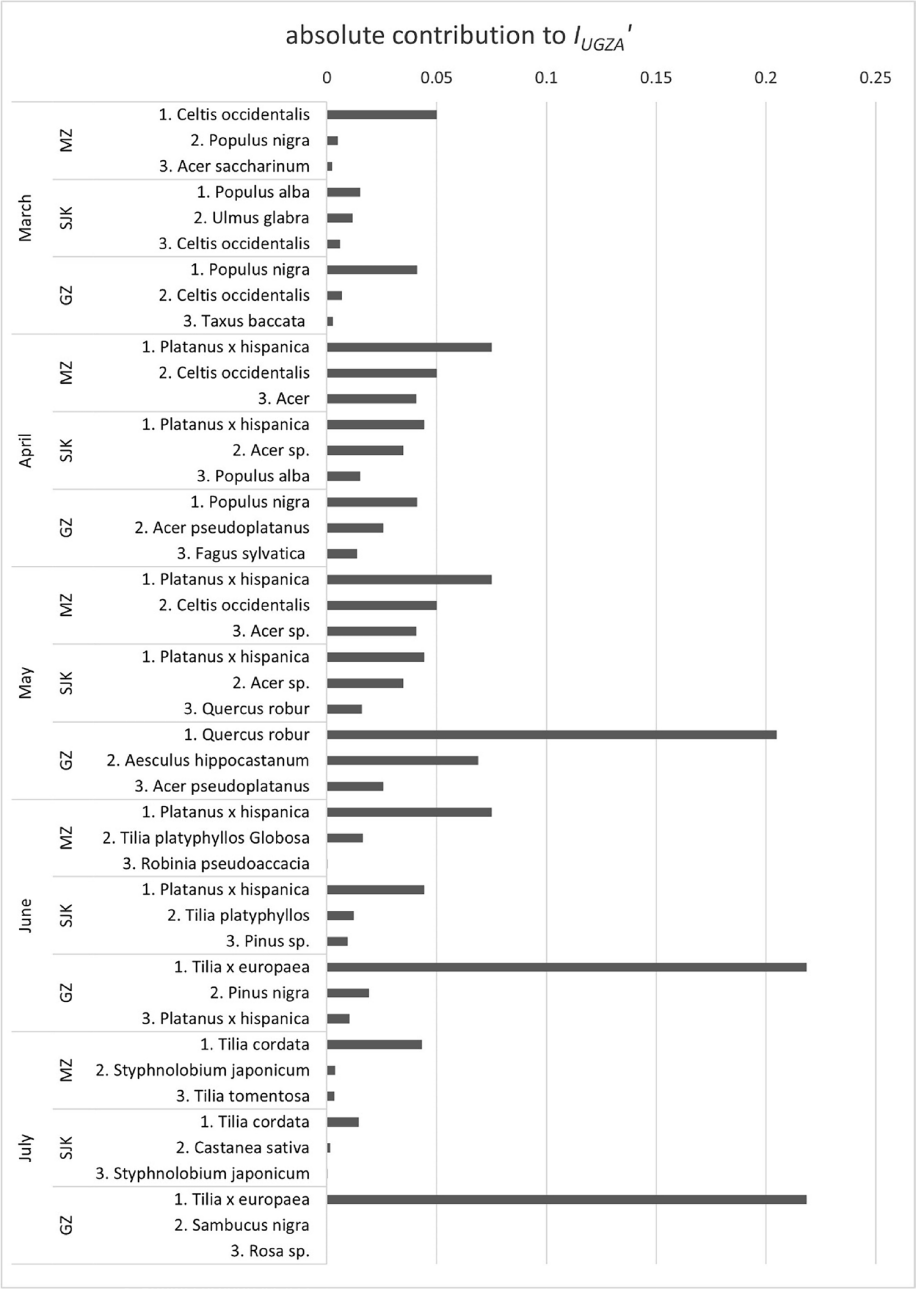
The principal contributors to the long-term allergenic potential in the studied parks: MZ — Medic Garden, SJK — Janko Kráľ Park and GZ — Grassalkovich Garden in individual months and their absolute contribution to the *I*_IUGZA_′ value

**Fig. 5:**
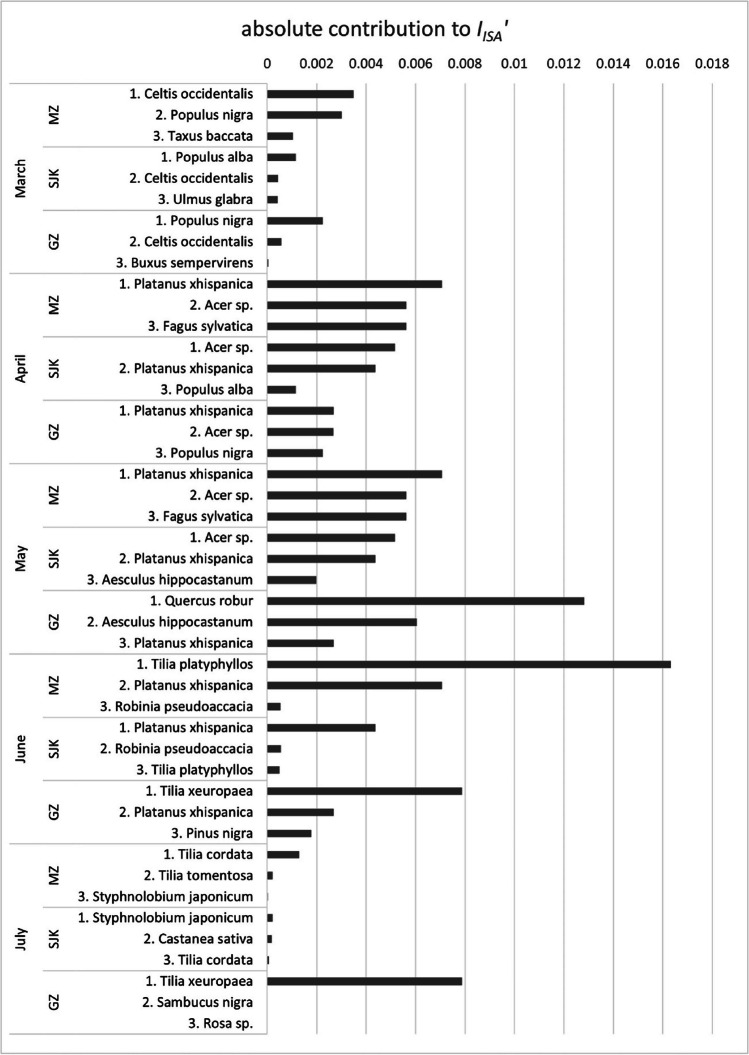
The principal contributors to the actual allergenic potential in the studied parks: MZ – Medic Garden, SJK — Janko Kráľ Park and GZ — Grassalkovich Garden in individual months and their absolute contribution to the *I*_ISA_′ value

An analysis of the greatest contributors to allergenic potential in the studied areas overall and in individual months are in Tables 3 and 4 and Figs. 4 and 5. Table 3 shows the taxa that would be top contributors to the long-term allergenic potential in the studied area when reaching their maximum potential size, which is a crucial information for future planning, represented by *I*_UGZA_′. Table 4 shows the actual allergenic potential in the studied area at present, represented by *I*_ISA_′. This information is most valuable for people with pollen-related allergies. If we would consider all studied parks as one area, the greatest contributors to both *I*_UGZA_′ and *I*_ISA_′ are the genera *Platanus*, *Acer* and *Tilia*.

**Table 3:** The principal contributors to allergenic potential in the studied parks in Bratislava: MZ — Medic Garden, SJK — Janko Kráľ Park and GZ — Grassalkovich Garden with their potential allergenic value (*PAV′*), number of specimen (*n*) in each area and the contribution to running and overall *I*_UGZA_′

Park	Taxon	*PAV′*	*n*	Contribution to *rI*_UGZA_ in %					Contribution to* I*_UGZA_′ in %
				March	April	May	June	July	
MZ	*Platanus × hispanica*	27	15	0	33.58	31.63	81.71	0	33.6
	*Celtis occidentalis*	27	15	79.37	22.39	21.09	0	0	22.4
	*Acer* sp.	12	26	0	18.19	17.14	0	0	12.13
	*Fagus sylvatica*	24	6	0	17.91	16.87	0	0	11.95
	*Tilia cordata*	6	13	0	0	0	0	85.73	6.47
SJK	*Platanus × hispanica*	27	65	0	34.16	28.78	64.17	0	32.5
	*Acer* sp.	12	163	0	26.77	22.55	0	0	16.98
	*Ulmus glabra*	27	17	29.37	8.93	7.53	0	0	8.5
	*Populus* sp.	18	25	38.01	11.56	0	0	0	7.33
	*Pinus* sp.	12	37	0	0	6.14	13.7	0	4.62
GZ	*Tilia × europaea*	12	64	0	0	0	87.67	99.99	44.4
	*Quercus robur*	12	36	0	0	56.61	0	0	20.81
	*Populus* sp.	18	8	80.39	39.5	0	0	0	8.32
	*Aesculus hippocastanum*	6	43	0	0	19.02	0	0	6.99
	*Acer* sp.	12	16	0	24.69	7.08	0	0	5.2
overall	*Platanus* × *hispanica*	27	82	0	32.13	23.84	48.72	0	26.56
	*Acer* sp.	12	205	0	25.1	18.63	0	0	13.83
	*Tilia* × *europaea*	12	64	0	0	0	25.35	55.38	9.21
	*Quercus robur*	12	58	0	0	18.74	0	0	6.96
	*Celtis occidentalis*	27	30	24.87	7.84	5.82	0	0	6.48

**Table 4:** The principal contributors to allergenic potential in the studied parks in Bratislava: MZ — Medic Garden, SJK — Janko Kráľ Park and GZ — Grassalkovich Garden with their potential allergenic value (*PAV′*), number of specimen (*n*) in each area and the contribution to running and overall *I*_ISA_′

Park	Taxon	*PAV′*	*n*	Contribution to *rI*_ISA_ in %					Contribution to *I*_ISA_′ in %
				March	April	May	June	July	
MZ	*Platanus* × *hispanica*	27	15	0	24.57	26.98	29.47	0	23.34
	*Tilia platyphyllos*	6	107	0	0	0	68.05	0	17.96
	*Acer* sp.	12	26	0	19.56	21.48	0	0	12.38
	*Fagus sylvatica*	24	6	0	19.55	21.48	0	0	12.38
	*Celtis occidentalis*	27	15	34.75	12.1	13.29	0	0	11.5
SJK	*Platanus* × *hispanica*	27	65	0	29.61	24.23	68.48	0	30.57
	*Acer* sp.	12	163	0	35.06	28.69	0	0	24.13
	*Populus alba*	18	25	39.69	7.89	0	0	0	5.43
	*Aesculus hippocastanum*	6	59	0	0	11.03	0	0	4.64
	*Quercus robur*	12	21	0	0	10.27	0	0	4.32
GZ	*Tilia* × *europaea*	12	64	0	0	0	61.18	99.98	25.88
	*Quercus robur*	12	36	0	0	45.69	0	0	21.08
	*Platanus* × *hispanica*	27	2	0	29.58	9.57	20.88	0	13.25
	*Aesculus hippocastanum*	6	43	0	0	21.58	0	0	9.96
	*Acer* sp.	12	16	0	29.54	9.56	0	0	8.82
overall	*Platanus* × *hispanica*	27	82	0	28.64	22.09	49.2	0	22.34
	*Acer* sp.	12	205	0	31.65	24.41	0	0	16.46
	*Tilia platyphyllos*	6	134	0	0	0	23.24	0	3.52
	*Quercus robur*	12	58	0	0	15.534	0	0	5.24
	*Tilia* × *europaea*	12	64	0	0	0	10.75	63.82	3.25

In MZ, the greatest contributors to the overall pollen-related allergenicity (both* I*_UGZA_′ and* I*_ISA_′) are the plane trees (*Platanus* × *hispanica*) flowering from April to June (Table 4). There are 15 of them, with both *tp* and *ap* = 3. In June, another big contributor to the actual allergenicity of the area (expressed by *I*_ISA_′) is the amphiphilous species *Tilia platyphyllos* with *tp* = 2 and *ap* = 3, since it is the most numerous species in the area (107 trees). These trees form an alley consisting of a globose cultivar of this species which does not reach the full size of the wild form, which is reflected in *I*_UGZA_′ where, despite its abundance, this particular cultivar is not among the principal contributors to its value. Thanks to its bigger potential size, which needs to be taken into account for future planning, this place belongs to *Celtis occidentalis*, with 15 specimen with *tp* and *ap* = 3, flowering from March to May.

In SJK, 16 specimens of *Platanus* × *hispanica* are also the greatest contributor to the allergenicity in the area from April to June (Table 4). 163 specimens of most abundant genus *Acer* with *tp* = 2 and *ap* = 3 cause another significant increase of allergenicity in April and May. These two taxa are the biggest contributors to both* I*_UGZA_′ and *I*_ISA_′, since most of their individuals are fully grown.

In GZ, 19 of the 34 identified taxa flower in May, causing a peak of allergenicity in this month (Table 4). The greatest contributors to both* I*_UGZA_′ and *I*_ISA_′ are 64 specimens of *Tilia* × *europaea* (*tp* = 2, *ap* = 3) flowering in June and July and 36 specimens of *Quercus robur* with *tp* = 3 and *ap* = 4, flowering in May.

Figures 4 and 5 show the principal contributors to the allergenic potential during the individual months in absolute numbers, which enable comparing the values between the studied areas. The most noticeable peaks in the total contribution to *I*_UGZA_′ are all in the GZ, caused by the flowering of *Quercus robur* in May and *Tilia* × *europaea* in June–July. However, most of these trees have been planted recently and will not reach their full size for several more decades. This is reflected in the absolute contribution to *I*_ISA_′, where the greatest peak is caused by the simultaneous flowering of 107 specimen of *Tilia platyphyllos* in MZ in May, although the peaks caused by the above mentioned species in GZ are also noticeable.

Table 5 shows the different parameters of the parks that can influence the values of *I*_UGZA_′ and* I*_ISA_′. The composition of species does not differ much between the three studied areas: from the 84 identified taxa, only 23 are unique for a single area, with most of them (17) in SJK due to its much bigger size. The composition of growth forms is also comparable, with the ratio of shrubs to trees between 1:4.38 in GZ and 1:5.21 in SJK. However, there is one significant difference: the density of woody plants. The values of *I*_ISA_′ best correspond with the density of trees, allergenic woody plants and wind-pollinated woody plants, while the values of *I*_UGZA_′ show negative correlation with Shannon’s biodiversity index.

**Table 5:** Chosen parameters of the studied parks in Bratislava: MZ — Medic Garden, SJK — Janko Kráľ Park and GZ — Grassalkovich Garden (bold type represents the greatest value)

	*I*_UGZA_′	*I*_ISA_′	Area (ha)	Number of woody plants	Number of woody plant species	Shrubs: trees ratio	Density of trees/ha	Density of allergenic woody plants (*ap* = 3–4)/ha	Density of wind pollinated woody plants (*tp* = 2–3)/ha	Shannon’s index
MZ	0.67	**0.091**	3.14	327	44	1:3.42	**80.57**	**79.3**	**7.64**	2.87
SJK	0.41	0.043	**23**	**890**	**65**	**1:5.21**	38.7	22.52	1.39	**3.57**
GZ	**0.985**	0.061	3.68	323	31	1:4.38	63.86	56.21	4.62	0.98

Spearman’s correlation between the values of *rI*_UGZA_, *rI*_ISA_ and several woody plant-related parameters of the parks in the individual months (Table 6) shows a significant positive correlation of *rI*_ISA_ with the number of flowering allergenic woody plants and their species in the given month, as well as with the number of tree and shrub species and density of flowering trees. Besides these, *rI*_UGZA_ also has a significant positive correlation with the number of flowering woody plants and their species in the given month, as well as the number of wind pollinated woody plants and their species. The two indices are also significantly correlated with each other.

**Table 6:** Spearman’s correlations between *rI*_UGZA_, *rI*_ISA_, and several woody plant-related parameters of the parks in the individual months (March–July)

	*rI*_ISA_	Number of flowering										Density of flowering trees/ ha
		Woody plants	Woody plant species	Allergenic woody plants (ap = 3–4)	Allergenic woody species (ap = 3–4)	Wind-pollinated woody plants (tp = 2–3)	Wind-pollinated woody species (tp = 2–3)	Trees	Tree species	Shrubs	Shrub species	
*rI*_UGZA_	0.927^***^	**0.588**^**+**^	0.636^*^	0.638^*^	0.716^*^	**0.588**^**+**^	0.689^*^	0.515	0.729^*^	0.442	0.701^*^	0.652^*^
*rI*_ISA_	×	0.503	0.503	0.638^*^	0.667^*^	0.503	0.542	0.406	**0.571**^**+**^	0.321	**0.585**^**+**^	0.732^*^

^+^*p* < 0.1; ^*^p < 0.05; ^***^p < 0.001

## Discussion

Our results show a difference of one magnitude order in using *I*_UGZA_, which takes into account the potential size that each species of woody plants in the study area can achieve, compared to *I*_ISA_ operating with the actual measurements of each specimen. The cause for this becomes evident when looking at the potential size of some tree species in the central European region. Several of the species can achieve height of 30 m and a 25-m diameter of the crown. However, if we look at the trees in urban parks, we see they achieve this size only rarely. The dendroflora in an urban environment has to deal with several stress factors (Wong et al. 2021), causing lowered life expectancy of trees. Even the planting plans of urban parks do not usually consider the potential size of trees under optimal conditions but plant them much closer to fill in the space in the early years of the tree’s development. As the trees mature in the time horizon of decades, such dense planting plans lead to the necessity to cut some of them to give the others more space to develop. For this reason, the difference between *I*_UGZA_ and *I*_ISA_ is especially noticeable in recently planted areas. In GZ, where most of the trees were planted in the last 30 years, *I*_UGZA_ is 16 times higher compared to *I*_ISA_. In SJK, it is 10 times higher and in MZ only 7 times. Jochner-Oette et al. (2018) calculated the values of *I*_UGZA_ = 0.173 and *I*_ISA_ = 0.018 in a public park in Eichstätt, Germany, showing a similar difference of one magnitude order between the two values.

When comparing the three studied localities, we see that SJK has the lowest value of both *I*_UGZA_′ (0.41) and *I*_ISA_′ (0.043). It is an English landscape park, with the highest area of the three studied parks, but lowest density of woody plants and lowest representation of highly allergenic species. The highest value of *I*_UGZA_′ (0.985) was found in GZ, while the MZ has the highest value of *I*_ISA_′ (0.091). We can conclude that the baroque garden, which is a more formal type of historical greenery, has a higher allergenic potential than the looser composition of an English landscape park. This is true even if the original composition of the area has been changed and modernised, like in MZ. The value of *I*_UGZA_′ in GZ is actually very close to 1, which would mean the same allergenic potential as an equal area with a full vegetation cover by fully grown anemophilous trees with highly allergenic pollen. When looking at the area, this is obviously not true: we can see ornamental lawns and paths lined with trees of small size, mostly young, but some even cut to maintain the shape. If all of these trees were fully grown to their potential size, their volume might even exceed the available space in the garden. In such a case of recently planted or renovated parks or highly ornamental historical gardens where trees are cut to maintain small shape, *I*_UGZA_′ can give a potential value that may never be achieved. It is a good tool for planning, but for comparing the actual allergenicity of the urban green spaces, *I*_ISA_′ should be considered despite the time-consuming process of taking the actual measurements of trees. For large areas, it may be possible to use approximate size categories while creating the dendrological inventory. The difference between *I*_UGZA_ and* I*_ISA_ also shows that using the potential size of trees in ideal conditions can skew its value into unrealistic numbers and therefore it might be better to use an average size the particular species achieves at maturity in urban conditions, considering the space limitations of the particular area. Building a database of these measurements for different climatic regions would be useful to achieve higher precision in the allergenic potential index used for planning of urban greenery.

However, a single value is not enough for people suffering from pollinosis to decide if they should avoid a certain area or not, and neither is it enough for management of the green areas. A situation is possible where the allergenicity in a certain area is exceptionally high during 1 or 2 months, but low values during the rest of the year push the potential allergenicity index lower and show no problem with the current composition of allergenic species. For this reason, we find it more informative to calculate a running allergenicity index for each month.

Based on a high *PAV* value, we can identify species that should be avoided in public spaces in general, but even taxa with a moderate *PAV* value, like *Acer* and *Tilia*, can cause a peak in allergenicity if their abundance is high. *rI*_UGZA_ makes it possible to plan the plantings and cuttings in the parks without causing such peaks of allergenicity in a particular month. Based on its values, we identified May as the month with highest allergenic potential in all studied parks, meaning that future plantings should avoid allergenic species that flower in this month and when cutting is needed, such trees should be considered before others. These include the species *Platanus* × *hispanica*, *Celtis occidentalis* and *Acer* sp. in MZ, *P.* × *hispanica* and *Acer* sp. in SJK and *Quercus robur* in GZ. However, in the case of SJK, we see the necessary individual approach to each park. The plane trees, contributing the most to the value of *rI*_UGZA_ here, are also some of the most valuable dendrological objects in the park. They are 200–250 years old and the biggest one has a circumference of 752 cm (Zahradníková et al. 2020). For this reason, future plantings should avoid trees that flower in the same months, and if cutting is necessary, it should be focused on younger individuals or on *Acer* sp.

The values of *rI*_ISA_ give information about the actual allergenic potential of the parks based on the actual measurements of the woody plant. Thanks to them, the public can make an informed decision about visiting parks and doing activities in them in the particular time of the year. The individual parks differ not only by the values of *rI*_ISA_, but also by the period of increased allergenic potential, making some more suitable for allergy sufferers than others. In MZ, the highest value of *rI*_ISA_ (not just in this park but from all of the studied locations) was rescored in April, but the period of increased allergenic potential lasts until June, caused first by the flowering of *Platanus* × *hispanica* and later *Tilia platyphyllos*. This makes MZ the least suitable for outdoor activities for pollen-related allergy sufferers from the studied parks. The second highest peak in *rI*_ISA_ was recorded in GZ in May, caused by the flowering of *Quercus robur*, which is one of the main allergens in the area (Hrubiško 1998). However, this is the only month of increased allergenic potential in this park, and so it is more advisable to visit it in the other parts of vegetation season, at least until the linden trees are fully grown and the values in June and July become higher, as we see by the values of *rI*_UGZA_. In SJK, *rI*_ISA_ is constantly lowest from all studied parks, with the exception of April. The period of increased allergenic potential lasts from April to May, so sensitive individuals should avoid this park in this period, but overall it is the best choice for outdoor activities during the year. Even in April and May, the particular allergenic species (again predominantly *P.* × *hispanica*) can be avoided due to the big size of the park.

As has been mentioned before, the pollination period can differ between years and so can the beginning and end of the phenophases, with an observed shift towards earlier timing with the ongoing climatic change (Cho et al. 2017), so the running allergenicity indices may not be fully accurate each year, but still offer a good approximation of the actual allergenicity for planning a visit to a particular location. However, it is important to note that the actual levels or airborne allergenic particles also depend on the weather, with highest values during a warm, sunny weather. Increased humidity can lead to lower release of pollen from anthers and faster sedimentation of pollen grains, but also to rupture of pollen caused by osmotic shock and releasing of small respirable allergenic molecules in the air (D'Amato et al. 2021).

Besides the pollen of woody plants, the pollen of grasses (Poaceae family) and fungal spores are the greatest contributors to the allergenicity in urban parks. Ornamental flowers usually grown in the parks are mostly entomophilic, and so their contribution to the allergenic potential is negligible. Grasses are considered one of the main aeroallergens, with *tp* = 3 and *ap* = 4 (Sabariego et al. 2021), and with flowering from May to August can considerably increase the allergenic potential of an area. However, the intensive mowing regime in public parks prevents flowering in most of the grasses that are a part of the lawns there. As such, the lawn maintenance purposedly aims to decrease the risk of pollinosis during the pollen season of grasses. Fungal spores are also abundant in urban green areas, especially those of the genus *Cladosporium* and *Alternaria*, reaching their peak in July–August (Kasprzyk et al. 2021). However, it is not possible to detect these without aerobiological sampling, which may not be available to the management of these areas. This is why we assume the contribution of fungal spores to be approximately equal in all parks and relatively low thanks to the immediate removal of dead plant matter (moved grass, woody plant cuttings and leaves, old declining trees) which is the primary source for fungal growth here. However, all of these factors would be necessary to consider in urban green areas with less intensive management.

Similarly to our results, Cariñanos et al. (2017) found a significant positive correlation between *I*_UGZA_ and the number of trees/ha and a negative correlation with Shannon’s biodiversity index. From the two indices we considered, we found *I*_ISA_ to be better correlated with the density of trees and *I*_UGZA_ with Shannon’s index (negatively). Regarding the running indices, *rI*_UGZA_ was better correlated with the parameters taking into account the number of flowering woody plants of particular type in the given month. However, these correlations can differ in various parks depending on the percentage of juvenile trees and site-specific growth conditions which may not allow some of the trees to reach their potential size even in maturity. The more the size of the trees in park nears their potential size, the closer these two indices will be.

Allergenicity indices have been used and tested in several regions of Europe, especially in the Mediterranean. Like with *I*_UGZA_ and* I*_ISA_, the running indices are applicable for determining the allergenic potential of urban green zones in other cities. However, when comparing the potential allergenicity of green spaces in different regions, it is necessary to consider local data in determining the *PAV* or every plant species and adjust the values in all indices accordingly, as well as adjust the maximum potential height of the vegetation in *I*_UGZA_. Aerobiological data and pollen calendars can help with determining whether a species is anemophilic or entomophilic, local phenological data give information about the time of pollination and allergological data are needed to determine the allergenicity of the species. This is especially important because the allergenicity of some species can differ in various regions based on their abundance, pollen production and sensibilisation of the population (Hrubiško 1998). When these local values are taken into account, the running indices provide an accurate information about the temporal variation of the allergenic potential in any urban green area.

## Conclusions

The allergenic potential of public green spaces varies during the vegetation period, and information about this variation is important for people suffering from pollinosis making a decision about their outdoor activities, as well as for the planning and management of green public spaces. Using a modified *I*_UGZA_ and* I*_ISA_ with calendar months of flowering instead of the duration of the pollination period, we created a running index, showing the variation of allergenic potential during the vegetation season in three important public parks in Bratislava. It enables to see the temporal of potential allergenicity during the year and identify the period of increased allergenic potential. The running indices are applicable and comparable for green areas in different locations, on the condition that the parameters determining the *PAV* of each species are checked and adjusted for the local conditions. Based on the identified woody plant species that most contribute to it, targeted measures can be implemented to reduce or avoid allergenicity within these areas. These measures may include selective plant removal, the introduction of low-allergenic species and strategic park design to minimise the dispersion of allergenic pollen, as well as avoidance of individuals sensitive to pollen of certain areas in particular months.

## Data availability

All data generated or analysed during this study are included in this published article.

## Abbreviations

MZ: Medická záhrada, Medic Garden
SJK: Sad Janka Kráľa, Janko Kráľ Park
GZ: Grasalkovičova záhrada, Grassalkovich Garden
*I*_UGZA_: Urban Green Zone Allergenicity Index
*I*_ISA_: Individual-Specific Allergenic Potential Index
*PAV*: Potential allergenic value of a given area
*ap*: allergenic potential of a given plant taxon
*tp*: type of pollination
*ppp*: length of pollen production period
*cmf*: calendar months of flowering
*I*_UGZA_′: Urban Green Zone Allergenicity Index calculated with *cmf* instead of *ppp*
*I*_ISA_′: Individual-Specific Allergenic Potential Index calculated with *cmf* instead of *ppp*
*PAV*′: potential allergenic value of a given area calculated with *cmf* instead of *ppp*
*rI*_UGZA_: Running Urban Green Zone Allergenicity Index
*rI*_ISA_: Running Individual-Specific Allergenic Potential Index
